# Assessment and abatement of the eco-risk caused by mine spoils in the dry subtropical climate

**DOI:** 10.1007/s10653-021-00885-3

**Published:** 2021-04-09

**Authors:** Alexey V. Alekseenko, Carsten Drebenstedt, Jaume Bech

**Affiliations:** 1 Department of Geoecology, Saint Petersburg Mining University, 2, 21st Line V.O., Saint Petersburg, Russian Federation 199106; 2 Technische Universität Bergakademie Freiberg, Freiberg, Germany; 3 University of Barcelona, Barcelona, Spain

**Keywords:** Mine dumps, Marl, Air pollution, Debris flow, Land reclamation, Mine rehabilitation

## Abstract

The highly rugged mountainous land topography of the Novorossiysk industrial agglomeration (NW Caucasus, Krasnodar Krai, Russia) and arid climate limit the restoration abilities of disturbed mine lands. Abandoned waste-rock dumps of a marl quarry occupy an area of ca. 150,000 m^2^ next to the cement plant, residential districts, and a commercial seaport. To assess the eco-risk, topsoil horizons of urban and mine-site Technosols and background Rendzinas were sampled and analyzed; measurements of particulate matter fractions PM1, PM2.5, PM4, and PM10 were conducted throughout the agglomeration. Fugitive dust emission from the unreclaimed marl dumps raises the PM2.5 content in the air by a factor of 2.68 on average. The high sorption capacity of the fine eluvium results in the accumulation of urban emissions by the dust and contributes to the subsequent soil pollution; the Cumulative Pollution Index of pedochemical anomalies reaches the high-risk level over the areas of up to 5 km^2^. Environmental threats caused by the mine dumps can be assessed more reliably by means of land zoning based on accumulated environmental damage indicators and the debris flow and waterspout risk calculation. To abate the technogenic impact caused by the mine spoils, reclamation actions must be taken including soil stabilization on sensitive sites by application of geosynthetic cover, hydroseeding of the mixture of soil improvers and seeds of herbaceous plants on the slopes, and anti-erosion plantation of cades (*Juniperus oxycedrus* L*.*) and smoke trees (*Cotinus coggygria* Scop*.*) at subhorizontal surfaces.

**Supplementary Information:**

The online version contains supplementary material available at 10.1007/s10653-021-00885-3.

## Introduction

### Disturbance and reclamation of a mining landscape

The mining industry significantly affects the environment due to pollution of air, soil, and surface and groundwater, landscape degradation, and damage to biodiversity, ultimately leading to economic damage (Opekunova et al., 2020; Rikhvanov et al., 2013; Sheoran et al., 2008; Timofeev et al., 2020). Russian mining and processing industries produce over 2 billion tons of solid industrial waste annually (Pashkevich et al., 2019). Residuals of extraction and processing of building materials are among the most capable of migration due to the high content of fine particles and lack of reclamation actions in dump-site areas (Nevskaya et al., 2019). According to various estimations, mining facilities of cement factories produce 50 to 100 thousand tons of waste a year. The resulting dumps are characterized by several adverse environmental features, such as dusting with a lack of moisture, compaction, and relatively low content of organic substances. The dust blown away from dump sites poses a threat when it (i) enters human respiratory tracts; (ii) absorbs pollutants in the atmospheric air, which subsequently settle in the topsoil layer causing the rise of pollution rate on farmsteads; and (iii) migrates with the water runoff from land surface to water bodies (Moiseenko et al., 2018), affecting urbanized areas (Cheremisina et al., 2017; Hełdak & Bykowa, 2017; Strizhenok & Ivanov, 2017; Tabatabaee Moradi & Nikolaev, 2018). Thus, the annually published Federal Reports *on the State and Protection of the Environment in the Russian Federation* state that the cement production industry is a major pollution source in a number of cities.

The model of sustainable development of the mining industry requires the responsible land use and restoration of affected soils (Androkhanov et al., 2019; Cao, 2007; Danilov et al., 2017; Isakov & Barygina, 2017; Ivanov et al., 2018; Pashkevich & Petrova, 2016). In a wide sense, reclamation is a set of works aimed at restoring the productivity and economic value of disturbed lands, as well as improving environmental conditions in harmony with the benefits of society. The restoration of plant cover can lead to the achievement of the goals of stabilization, pollution control, visual improvement, and elimination of threats to humans. Soil provides the basis for these processes; therefore, its characteristics directly affect the future stability of the restored plant community. Long-term revitalization of mine-site soils requires the reestablishment of stable nutrient cycles for plant growth and microbiological processes (Kavamura & Esposito, 2010; Zen'kov et al., 2018). The restored terrain must fulfill one of the main tasks of environmental sustainability, that is, maintain the possibility of land use for future generations.

Laws and legislative decrees require mining companies to ensure environmental safety and return disturbed lands to an acceptable state. Nevertheless, some approaches to restoration of technogenic landscapes have shown their inefficiency, as they did not ensure the stable development of ecosystems and often cause environmental damage (Berger, 1990; Maczkowiack et al., 2012; Perminova et al., 2016). *The Land Code of the Russian Federation* states that entities and persons whose activities have led to pollution or soil disturbance are required to ensure their reclamation, i.e., to take measures preventing further land degradation and restore their fertility by bringing it into a condition suitable for use under the permitted purpose.

### Mine spoils in the dry subtropical climate

**Fig. 1:**
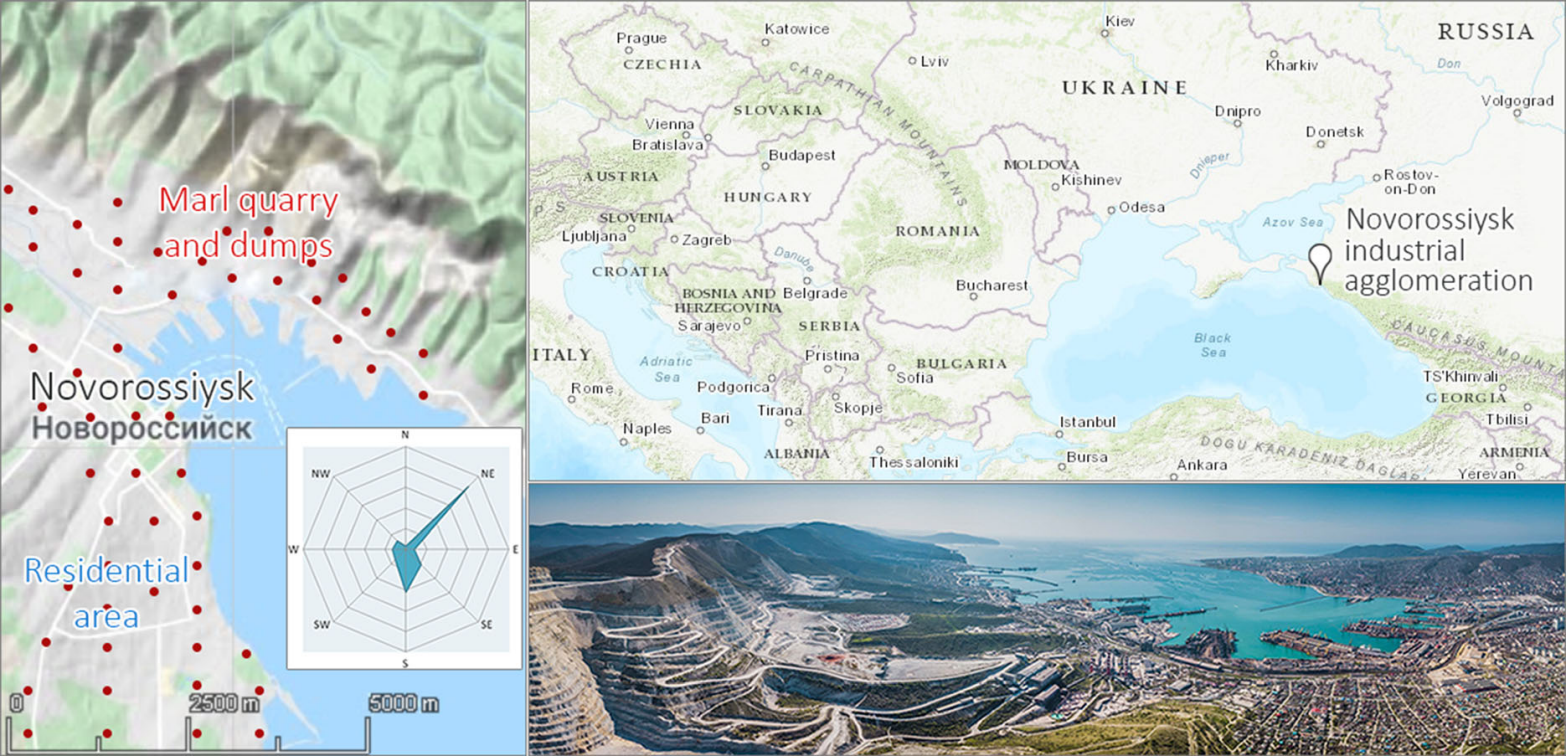
Study area, clockwise. Right upper block: geographic position (modified from the *Bing* map). Right lower block: aerial photograph image (credits: E. Sorokin) of the Markotkh Range, mined quarry, Tsemes Bay, and residential districts. Left block: land topography changing from the sea level to 558 m (modified from the *Google Landscape* map) and prevailing and dominant winds from the northeast; red points indicate sampling areas

A cement factory in the Novorossiysk industrial agglomeration (NW Caucasus, Krasnodar Krai, Russia) was put into operation after the discovery of one of the largest marl deposits in the world in 1882 (Fig. 1). Although the plant remains active, overburden mining operations were completed and non-reclaimed marl mine dumps were abandoned. The dry subtropical climatic conditions limit the landscape restoration chances and require man-aided revitalization to abate the eco-risk caused by mine spoils. A comprehensive environmental study of the industrial agglomeration was required for the identification of most hazardous sites, as well as for substantiation of the necessity of reclamation needed for the lowering of the technogenic burden of the mine dumps on the environment.

Issues of the landscape–geochemical exploration allowing quantitative assessment and delivery of practical advice on how to improve the ecological situation have been thoroughly examined, and a significant amount of environmental data are collected describing the impact of mining and processing industries, as well as practicable methods for rehabilitation of lands affected by the production of building materials among other things (Alguacil et al., 2006; Androkhanov et al., 2019; Doley et al., 2012; Kuter et al., 2014; Moreno-de las Heras, 2008; Pivnyak et al., 2011; Timofeev et al., 2014). However, the available assessment data obtained in the course of studies of territories affected by cement crude material mining include only concrete cases and contain no recommendations on how to handle environmental damage in other conditions beyond the limits of such cases. Thus, the study objective was to develop a system allowing: (i) efficient *assessment of the industry pressure* on adjacent areas based on the landscape–geochemical monitoring and (ii) *control of risk areas* based on land zoning depending on the terrain specifics by eco-friendly economically feasible reclamation measures.

## Materials and methods

### Study area

The territory of the city of Novorossiysk is in the Alpine–Himalayan belt, on the folded base of the platform, deformed at the end of the Oligocene–Miocene. The studied area consists almost exclusively of sedimentary rocks, which undergo a folded dislocation since the Tertiary era. The urban area on the Black Sea coast has a *highly rugged mountainous land topography*, which main morphological element is the Markotkh Range, the northwestern tip of the Greater Caucasus, as well as the Abrau Peninsula. In the vicinity of Novorossiysk, the mountain ranges are composed of Upper Cretaceous sediments that include calcareous light blue shale clays, marls, and layered sandstones, and siliceous steel-gray limestones. Due to the low thickness of the overburden, the development of a marl deposit for cement production is carried out in an open way on the slopes of the Markotkh Range. The trace element composition of marls and the soils formed on them in undisturbed forest landscapes of the Northwest Caucasus are presented in Table 1 alongside the data on the studied technogenic landscape.

**Table 1:** Trace elements in parent rocks of the Markotkh Range at the northwestern tip of the Greater Caucasus and soils of the background (Alekseenko et al., 2008) and urban areas (mg/kg)

Elements	Marls	Background Rendzinas	Technosols					
	Mean	Mean	Max	Min	Median	Mean	STD	CV (%)
Ba	433.30	800.00	3000.00	300.00	1000.00	940.00	463.28	49.28
Co	7.30	21.00	50.00	10.00	20.00	20.54	7.45	36.29
Cr	53.30	137.00	150.00	50.00	80.00	77.38	16.98	21.94
Cu	30.00	58.00	300.00	40.00	60.00	75.54	43.16	57.13
Li	40.00	52.00	60.00	30.00	50.00	50.00	7.29	14.58
Mo	1.30	2.60	6.00	0.60	2.00	2.12	0.88	41.27
Ni	20.00	48.00	60.00	30.00	50.00	46.00	8.44	18.35
Pb	18.30	42.00	600.00	20.00	50.00	69.23	84.90	122.63
Sn	2.30	5.10	20.00	3.00	5.00	5.18	2.26	43.52
Sr	6.70	340.00	1500.00	400.00	800.00	875.38	172.33	19.69
V	30.00	143.00	150.00	30.00	60.00	69.23	16.71	24.13
Zn	70.00	123.00	2000.00	60.00	150.00	214.00	255.15	119.23

**Fig. 2:**
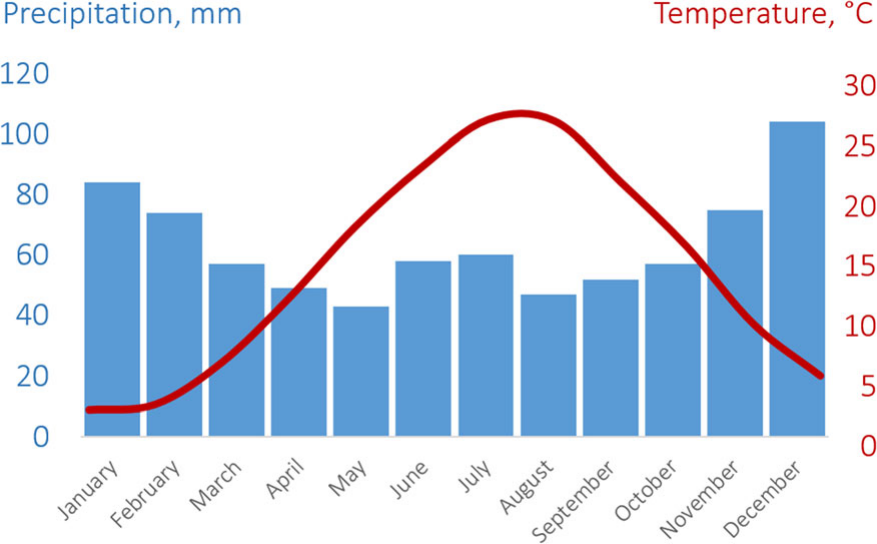
Climatic conditions of Novorossiysk city (based on the *worldclimate.com* source data)

Climatic features significantly impact the migration and concentration of chemical elements in the region. The city is in a dry subtropical climate, characterized by *dry hot summers and humid warm winters* (Fig. 2). Exposure to Siberian and Azores anticyclones leads to stable clear weather, cold in winter and warm in summer. The passage of cyclones from the Atlantic region or the Mediterranean Sea brings rainy, unstable weather. The annual temperature amplitude is about 20–25 °C. The average temperature of the coldest month, January, in Novorossiysk is + 3 °C. The average temperature in July on the Black Sea coast ranges from + 23 to + 25 °C, often reaching + 35 °C. The average annual temperature in the city exceeds + 10 °C. The annual rainfall in Novorossiysk averages at least 700 mm; stable snow cover is not formed.

Juniper woodlands are developed within the undisturbed dry marl slopes on the Black Sea coast of the Caucasus. Oak and oak–hornbeam forests grow on the watershed parts, giving way to beech and chestnut on the eastern slopes. Background *Rendzinas* or *Leptosols* are humus-rich shallow soils formed from the carbonate-rich parent material. The studied territory of the Novorossiysk agglomeration is characterized by anthropogenically disturbed poorly textured *Technosols* and *mine spoils* characterized by low humus content and a high percentage of technogenic inclusions.

*Strong winds* of the northeast and south directions (Fig. 1) are among the main meteorological features of the city of Novorossiysk and the adjacent territories. On average, about 20 days a year, the wind speed exceeds 15 m/s and the katabatic wind *bora* rushes down the Markotkh Range at hurricane speeds 46 days a year. The highest *bora* frequency is in the winter months (an average of 18 days per season), as well as autumn (12 days) and spring (11 days).

The atmospheric environment and geomorphological conditions contribute to the formation of *debris flows*. They are particularly destructive if not only excessive rainfalls but also tornadoes contribute to their formation. Temperature drop on the coastal line contributes to the formation of intense columnar vortexes over the Black Sea. Sometimes, such *waterspouts* move by land, at a distance of tens of kilometers. In Novorossiysk, in recent years this happened in the Shirokaya Balka resort area in 2002 and 2018 when the seawater discharge in a gully led to a rather large catastrophe with the destruction of buildings and human casualties. With this, not only demolition occurs, but the environmental situation changes as well; specifically, the geochemical features of soils are altered when water with a high salt content comes to land from the sea. Having much in common with the inundation aftermath, but often to a much greater extent, debris flows transform the geochemical patterns of soils and, in particular, the content of metals.

### Sampling and analytical procedures

A field study of the Novorossiysk industrial agglomeration, including territories adjacent to the marl quarry and dump sites, was carried out during the summer seasons of 2014 and 2015. Threefold repeated field measurements of dust fractions *PM1, PM2.5, PM4,* and *PM10* in the air were conducted using DustTrak 8533 (*TSI*, USA). The measurements were carried out at a height of 1.5 m at 46 points on a regular grid with a step of 2 km. *The topsoil horizon* (0–5 cm) was sampled on 82 plots using the composite sample technique: On a plot of approximately 10 m^2^, the soil was taken from test pits at least three times and placed in one plastic bag; their total weight was about 3 kg. Under laboratory conditions, mixing and averaging were performed. Sample preparation for the determination of chemical element contents, pH value, and particle size distribution was completed in the Common Use Centre of the Saint Petersburg Mining University.

*The particle size distribution* was determined in the range from 10 nm to 3 mm with a measurement error of 0.6% on an LA-950 laser analyzer (*Horiba*, Japan). *Active soil acidity* was studied by a potentiometric method in a suspension with a ratio of soil mass to water volume equal to 1:5 using a combined pH electrode. In 17 samples from soil pits, *the organic carbon content* was determined by the thermal spectral method on a TOC instrument (*Shimadzu*, Japan).

Concentrations of 53 *trace elements* were determined on an inductively coupled plasma optical emission spectrometer ICPE-9000 (*Shimadzu*, Japan). Spectral emission evaporation analysis was performed for the range of contents from n⋅10% to n⋅10^–4^% on the DFS-8-1 spectrometer (*Spectral Laboratory*, Russia). Internal laboratory control was carried out by re-analyzing samples sent under encrypted titles. The control samples made up 4% of their total number. After obtaining the results of the analyses, the convergence of the ordinary and control samples was checked and confirmed statistically. To categorize geochemical associations of major pollutant elements in the soil, *the Cumulative Pollution Index* (CPI) was calculated, classifying the environmental hazard of increased concentrations (Timofeev et al., 2014). For this purpose, when calculating CPI, world abundances were used as a reference, rather than local background element concentrations, in some cases naturally exceeding safe levels: CPI = *∑ *(*EF* × *TF*) *−* (*n* *−* 1), where

- *EF* stands for an enrichment factor, calculated as proposed by N.S. Kasimov and D.V. Vlasov (Kasimov & Vlasov, 2015) relative to the abundances of Mo and Ba (Rudnick & Gao, 2003), Bi, Co, Cu, and V (Hu & Gao, 2008), Sn (Wedepohl, 1995), As, Cr, Ni, Pb, Sr, W, and Zn (Grigoriev, 2009) in the upper continental crust;
- *TF* is the toxicity factor equal to 1.5 for elements of the I hazard class (Table 2), 1.0 for the II-class elements, and 0.5 for the III-class and non-hazardous elements (Guidelines, 1999);
- *n* is the number of accumulated chemical elements having EF > 1.3;

**Table 2:** Hazard classes (HCs) and toxicity factors (TFs) of the chemical elements (CE)

CE	As	Bi	Cd	Co	Cr	Cu	Li	Mo	Ni	Pb	Sb	Sr	V	W	Zn	Ba	Mn
HC	2	2	2	2	3	3	2	2	3	2	2	2	3	2	3	2	3
TF	1.0	1.0	1.0	1.0	0.5	0.5	1.0	1.0	0.5	1.0	1.0	1.0	0.5	1.0	0.5	1.0	0.5

The obtained CPI values in soils were ranked by hazard level of pollution (Table 3).

**Table 3:** Soil contamination degrees and corresponding safety categories (Saet et al., 1990)

Contamination and environmental threat level	Cumulative Pollution Index
Allowable, low hazardous	< 16
Average, moderately hazardous	16–32
High, hazardous	32–64
Very high, very hazardous	64–128
Maximum, critical	> 128

The eco-risk of soil and air pollution was assessed by using the health and hygiene standards of maximum permissible concentrations (Tables 4, 5).

**Table 4:** Maximum permissible concentrations (MPC) used as soil sanitary standards (GN 2.1.7.2041-06; GN 2.1.7.2042-06) (mg/kg)

Element	As	As	Cd	Cu	Hg	Ni	Pb	Pb	V	Zn
MPC	2.0	10	2.0	132	2.1	80	32	130	150	220

**Table 5:** Maximum permissible concentrations (MPC, mg/m^3^) of suspended solids in the air of urban and rural settlements (GN 2.1.6.3492–17)

Substance	MPC		Hazard class
	Maximum single dose	Daily average dose	
PM^(a)^	0.50	0.15	3
PM10^(b)^	0.30	0.06^(c)^	–
PM2.5^(d)^	0.16	0.035^(e)^	–

(a) The compositionally undifferentiated dust (aerosol) contained in the air of settlements, not considering separately the aerosols of organic and inorganic compounds (metals, their salts, plastics, pharmaceuticals, etc.); (b) mean year concentration is 0.04 mg/m3; (c) 99 percentile; (d) mean year concentration is 0.025 mg/m3; and (e) 99 percentile

Following a uniform sampling scheme, the percentage of the area of contaminated soils was determined as the number of points with MPC exceeded for individual elements, referred to a total number of sampling points in the territory of the industrial agglomeration.

## Results and discussion

### Are the mine spoils hazardous?

#### Geotechnical characteristics

**Fig. 3:**
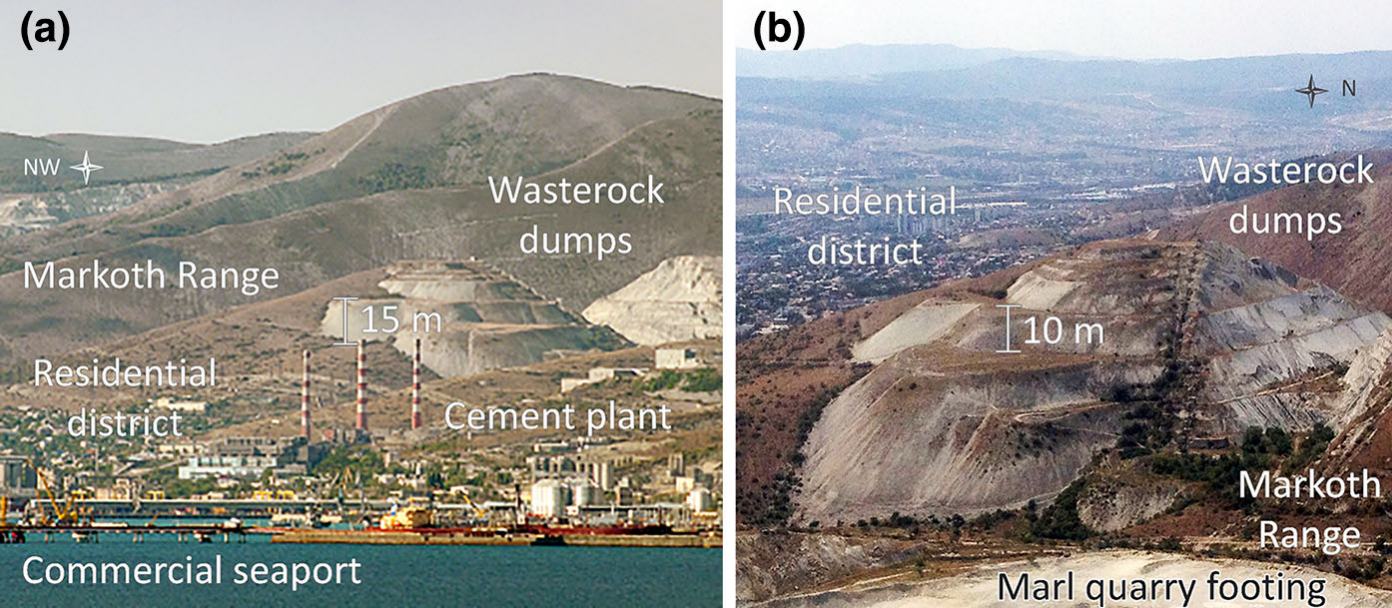
Overburden marl mine spoils: **a** general layout within the Novorossiysk industrial agglomeration (credits: E. Ptushka/Strana.ru) and **b** disposition of the dumps at the slopes

The waste of overburden and substandard marl generated during the development of marls used in cement production since the 1960s were transported by dump trucks to two external dumps on the slopes of the Markotkh Range at heights of 100–150 m above the Tsemes Bay (Fig. 3). At present, dumping work is not being carried out in the quarry, since the required volume of overburden has been removed. Plateau-shaped terraced dumps have a relative height of ca. 50 m; the length and width of both waste storages differ slightly. The total area of mine spoils is approximately 150,000 m^2^, of which about 105,000 m^2^ are 15–45° *steep slopes*, while *subhorizontal terraces* occupy about 45,000 m^2^. The slopes with a steepness of more than 15° are unsuitable for intensive land use but may be suitable for reforestation and grazing. Subhorizontal dumpsites include boulder clusters, as well as pits and ditches that impede the vegetation restoration. Besides, wide leveled surfaces with a slope of less than 2° can undergo seasonal waterlogging, which leads to the development of anaerobic conditions, detrimental to plants.

Soil particles of less than 2 mm diameter contribute to the accumulation of water and nutrients in the soil. Soils consisting of coarse material cannot hold enough water available for plants to maintain active growth during the summer months (Moradi et al., 2018). The content of large fragments of marl in the upper 50 cm of the studied dumps varies from 50 to 70%. The particle size distribution analysis of the remaining regolith revealed that sandy loamy (particles of d < 0.01 mm make up 10–20%) and loamy (20–45%) fractions are prevailing. This particle size distribution is acceptable for seedling growth during reclamation since a larger proportion of the sand fraction does not accumulate a sufficient amount of moisture and nutrients, unlike loams and clays. Excessive clay content can also impede plant cultivation due to the risk of surface crust formation. As marl weathers, the relative proportion of fine fractions increases, but deflation and erosion limit the accumulation of fine earth. The study in the Mediterranean arid region, close in terms of conditions to the area of Novorossiysk (Moreno-de las Heras et al., 2008), showed that bulk masses with a coarse-grained material content of more than 50% have a weak natural restoration potential and require man-aided reclamation. Thus, dumps remain almost bare after 50 years due to, among other things, excessive stoniness of the substrate. However, particle size distribution on flat dump sites allows reclamation without additional soil application.

Soil aggregates control the erosion potential and moisture reserves and affect the availability of nutrients and accumulation of organic carbon. The compaction occurring during the formation of the dumps reduces the moisture capacity and impairs aeration. Field texture determination of the surface horizon of dumps showed that silty (with a cube edge < 0.5 cm) and finely crumbly (from 0.5 to 1 cm) aggregates made up the major part of the Technosol. This makes the material unstable and the dumps are subject to wind blowing and surface water washout. The formation and stability of soil aggregates depend on organic substances and microorganisms involved in the binding of soil particles. Consequently, the cultivation of plant communities is necessary to improve soil aggregation, to raise moisture and nutrient reserves, and to prevent erosion on the dumps.

In conditions of arid climate in the Novorossiysk region, the average moisture content in the surface horizon of dumps during the winter period is about 5% and about 2–3% in the summer (Kazeev & Kolesnikov, 2015), what is insufficient for seedlings to grow (Maiti, 2013). In this regard, in the first year after reclamation, additional watering of plants will be required. The use of wheeled and tracked mining machines led to soil compaction (Jordán et al., 2016) to the bulk density of > 1.7 g/sm^3^, whereas the density of productive natural soils usually ranges from 1.1 to 1.5 g/sm^3^ (Pivnyak et al., 2011). Compaction of loose deposits directly limits plant growth, as most species cannot spread roots effectively. Also, the volume of retained moisture required for plants during the drought period is reduced, especially in the weakly weathered coarse rocks. Compacted horizons can become technogenic aquicludes during the rainy season, causing saturation with moisture and the formation of anaerobic gleyic conditions in the root propagation zone. In the conditions of an arid Black Sea climate, the presence of loose unconsolidated sediments with a thickness of at least one meter is necessary to maintain the water supply of plants in the summer. When designing restoration works, these conditions dictate the need for the selection of woody plants that tolerate dry seasons and have a root system adapted to stony soil substrates.

#### Geochemical characteristics

Acidity is the most frequently used indicator of the quality of mine spoils, as its level of the surface horizon can quickly change with the supergene oxidation. In the case of the Novorossiysk agglomeration, an increase in pH is due to the weathering of rocks containing Ca/MgCO_3_. A study of the pH of the dumps showed average values of 8.6–8.7 in the surface horizon, with growth to a level of 9.2 at the depth of 20–40 cm. The weathered carbonates make up 50–75% of the marl and majorly contribute to this value. Accordingly, the acid–alkaline conditions of the dumps do not require amendment.

Since the thickness of the fertile horizon layer was less than 10 cm, its removal was carried out in a bulk manner, without separation of the underlying bedrock and soil. In the conditions of the dry subtropical climate, with the main volume of precipitation falling in the winter, maintaining the *soil fertility* seems to be problematic, as the number of anaerobic bacteria increases in dumps at a depth of about 1 m during the rainy season, affecting the geochemical. Given the long-term storage period of the fertile soil layer with overburden and substandard marl, permanent deterioration of soil properties can result in an almost complete loss of its biological productivity in the future if no land reclamation measures are taken. The organic carbon content of more than 0.75% indicates the possibility of plant cultivation under the local landscape conditions (Kazeev & Kolesnikov, 2015). The established C_org_ content in the surface horizon of dumps varies from 0.35 to 1.85%. In addition, Fe, Mn, Cu, and Zn are among the metals necessary for plant growth; rock weathering provides these elements in the soil in a form accessible to plants. Reclamation can be considered environmentally sustainable at the following concentrations of elements in the soil: Fe > 4.5 mg/kg, Mn > 1.0 mg/kg, Zn > 1.0 mg/kg, and Cu > 0.4 mg/kg (Barcelo & Poschenrieder, 2003; Schmidt, 2003). Analytically determined gross concentrations of Fe, Mn, Cu, and Zn indicate a potential supply of the metals necessary for plants (Table 6). Given the significant content of coarse material and the relatively low content of the fine fraction in the industrial soils of the dumps, it is necessary to select types of herbaceous plants that ensure the accumulation of nutrients in the soil, which will limit fertilizer application and create a sustainable ecosystem.

**Table 6:** Bulk levels of Fe, Mn, Cu, and Zn in the upper horizon of the mine spoils

Substance	Fe_2_O_3_ **(**%**)**	MnO (%)	Zn (mg/kg)	Cu (mg/kg)
Average content	4.35	0.12	281	91

*Trace element composition* of weathered marl and its debris is analogous to the undisturbed uncontaminated background Rendzinas typical of the Northwest Caucasus. Hence, the unreclaimed dumps formed in the Novorossiysk industrial agglomeration, mainly consisting of large rock fragments with an admixture of loose deposits devoid of organic matter and nutrients, are geochemically safe but are potentially dangerous to the environment through the deflation and erosion.

### Risk assessment

#### Pollution intensification risk

Fine particulate matter with an aerodynamic diameter of ≤ 1 μm (PM1), ≤ 2.5 μm (PM2.5), and ≤ 10 μm (PM10) is principal in environmental studies (O'Dowd et al., 2002; Radomskaya et al., 2018). The air pollution study in the Novorossiysk industrial agglomeration (the triplicate measurement results are provided in the Supplementary section) showed that the average content of *the sum of PM1–PM10* was 0.594 mg/m^3^ at the dumps and in their immediate vicinity (at a distance of up to 100 m); the peak average concentration reached 2.680 mg/m^3^. The average content of the sum of the same fractions was 0.060 mg/m^3^ in the air of the undisturbed territory northeast of the Markotkh Range; the maximum value was 0.132 mg/m^3^.

According to the World Health Organization, airborne PM2.5 content is currently considered the best indicator of environmental impact (WHO, 2014). After the long-term inhalation of air with a high content of particles with a diameter of fewer than 2.5 μm with alveolar deposition in the human body, a number of diseases progress, and an average life expectancy is reduced by about 1 year (Defra, 2008). The average *PM2.5* content in the dump area (0.134 mg/m^3^) is more than 2 times higher than the average values typical for background areas (0.050 mg/m^3^). These results are consistent with averaged data showing that the total PM2.5 concentration is about 2 times higher in urban air than in rural areas (Pöschl, 2005).

Initial fugitive dust emission takes place at the dumps on the hillside of the Markotkh Range (Fig. 3). Dust measurement at the relative height of 1.5 m showed that in case of prevailing northeast downslope winds on the leeward slopes (including the Novorossiysk bora windstorm), the soil is mechanically eroded and fine particles (< 10 µm) are blown off the bare surface. Migrating with the wind, solid particles are carried toward the western side of the Tsemes Bay, which leads to an increase in the concentration of the sum of fractions by 2.5–3.0 times in the central and residential areas of the agglomeration in comparison with the background.

In urban landscapes, aerosols react with other impurities (Karagulian et al., 2015). The formation of new aerosols in air occurs at an average rate of up to 100 particles per 1 cm^3^ per 1 s; the increase in particle size due to the formation of bonds is from 1 to 25 nm per hour and the process proceeds much more intensively during the summer than in the winter (Kulmala et al., 2004). Chemical reactions occur both on the surface and within solid and liquid aerosol particles and can affect the chemical composition of the gas phase in the atmosphere, as well as the properties of atmospheric particles and their effect on climate and human health. The interaction of the primary emitted dust particles with the chemicals contained in the atmospheric air leads to the formation of secondary polluted aerosols (Kasimov et al., 2020).

**Fig. 4:**
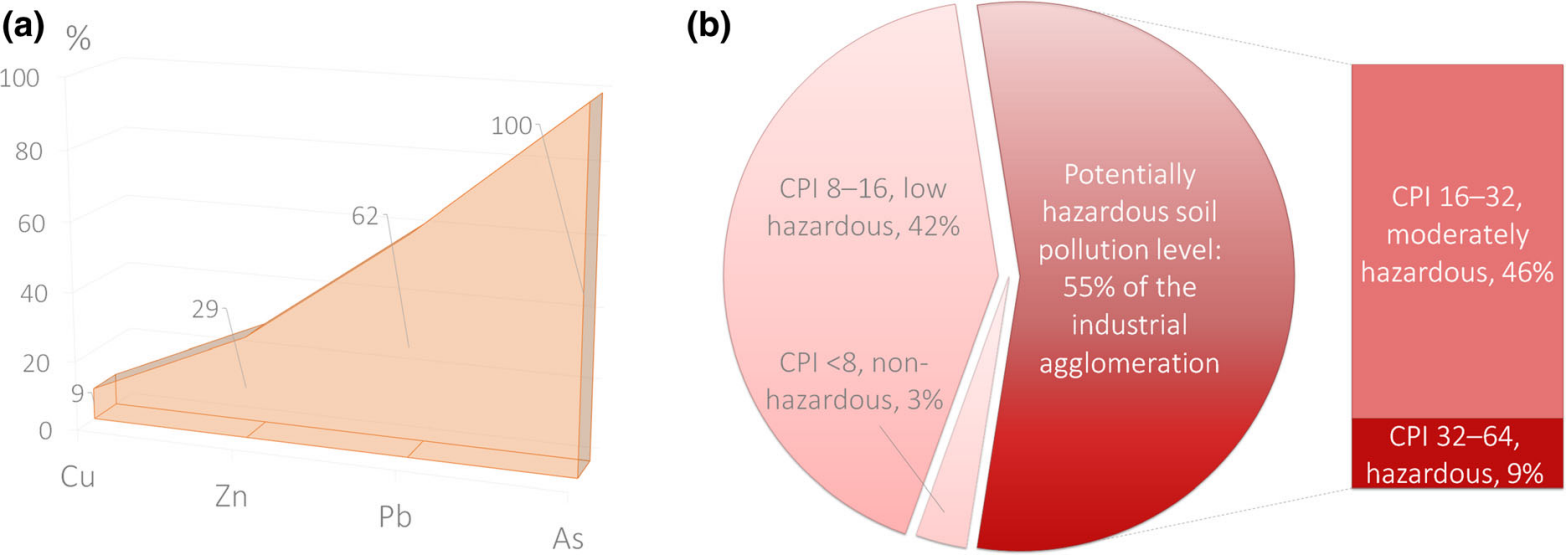
Proportional share of the agglomeration territory: **a** where the pollutant element contents exceed the maximum permissible concentrations in soils; **b** with varying levels of the Cumulative Pollution Index

The implication is that (i) the particles pose a risk of inhalation and (ii) having high sorption capacity, dust accumulates urban pollutants contained in the atmospheric air and subsequently precipitates on the topsoil. Since the continuous measurement of dust concentrations is challenging, the soil cover remains the utmost symptomatic indicator of the ecological state of landscapes as it is a depositing medium reflecting the whole anthropogenic impact (Luo et al., 2007). Its eco-risk ranking was performed by calculating the proportional share of the territory where the pollutant element contents exceed the maximum permissible concentrations in soils (Fig. 4a); the share makes up from less than 30% (Cu, Zn) to 62% (Pb) and 100% (As). The maximum exceedance of the state-established MPC reference standards makes up the association of As_50_Pb_19_Zn_9_Cu_2_, creating geochemical anomalies of up to 5 km^2^. Accumulation of Pb, Zn, and Cu in the urban soils can be explained by the air pollution from the cement plant and transport infrastructure, as well as the legacy contamination. Although the levels of As and the widespread exceeding of the permissible level reflect a possible threat to the environment and human health, its concentration is due to the increased regional background, which was noted in a comparison with respect to the abundance in the upper continental crust (Grigoriev, 2009).

The studied soil cover developed on marl and limestone bedrock creates favorable conditions for the accumulation of cations on carbonaceous geochemical barriers (Alekseenko et al., 2017; Perel'man, 1986). The fate of pollutants in the urban environment can be significantly transformed depending on changing geomorphological conditions and vegetation, leading to the redistribution of elements along the landscape–geochemical catena. Therefore, the calculation of the Cumulative Pollution Index was needed to assess the overall state of the soil affected by the dusting dumps. It showed that over 50% of the entire study area (ca. 30 km^2^) can be characterized as moderate eco-risk or high eco-risk in terms of pollutant element accumulation in its topsoil horizon (Fig. 4b). The environmental damage to the soil cover was calculated as *D*_total_ = *D*_pollution_ + *D*_waste_ + *D*_overlap_ = 11.83 + 58.5 + 106.47 =  176.8 million = €2.2 million. The state-adopted computational technique takes into account the following parameters: *D*_pollution_ = Ratio × Area × Depth × Land Use Tariff × Geographical Zone Tariff; *D*_waste_ = Σ (Waste Mass × Toxicity Factor) × Land Use Tariff; *D*_overlap_ = Area × Depth × Land Use Tariff × Geographical Zone Tariff.

#### Debris flow risk

The unfixed bare surface of the dumps and the impossibility of the vegetation self-restoration without special land improvement measures give the way to rain and wind erosion. We found that the mine soils pose an environmental threat being not only a source of dust but also an unstable mine-waste storage site, sensitive to debris flows. The unsuccessful junction of geomorphological, geological, and climatic conditions of the dumpsite can result in the situation when water-laden masses of loose rock rush down the dumps at the Markotkh Range toward residential and industrial districts.

##### Land topography

The southern slopes of the Markotkh Range are quite steep. The length of several gullies starting at the pass and crossing the highway and railroad, as well as part of the city itself, exceeds 4 km. The catchment area of the gullies exceeds 3 km^2^. This creates geomorphological prerequisites for the formation of debris flows, i.e., turbulent mud-stone movements of tremendous destructive power. Rock fragments usually make up to 50%, the water portion is up to 30%, and the loose sediments can give 20–40% of the flow volume (Barinov, 2010). In the Northwest Caucasus and especially the area of Novorossiysk, numerous debris flows resulted in catastrophic destruction with several casualties and enormous economic damage (Yefremov et al., 2010). However, geomorphological assumptions alone are not enough to determine the potential for debris flows. A sufficient volume of all the components that make up the mudstone stream is necessary.

##### Water

**Fig. 5:**
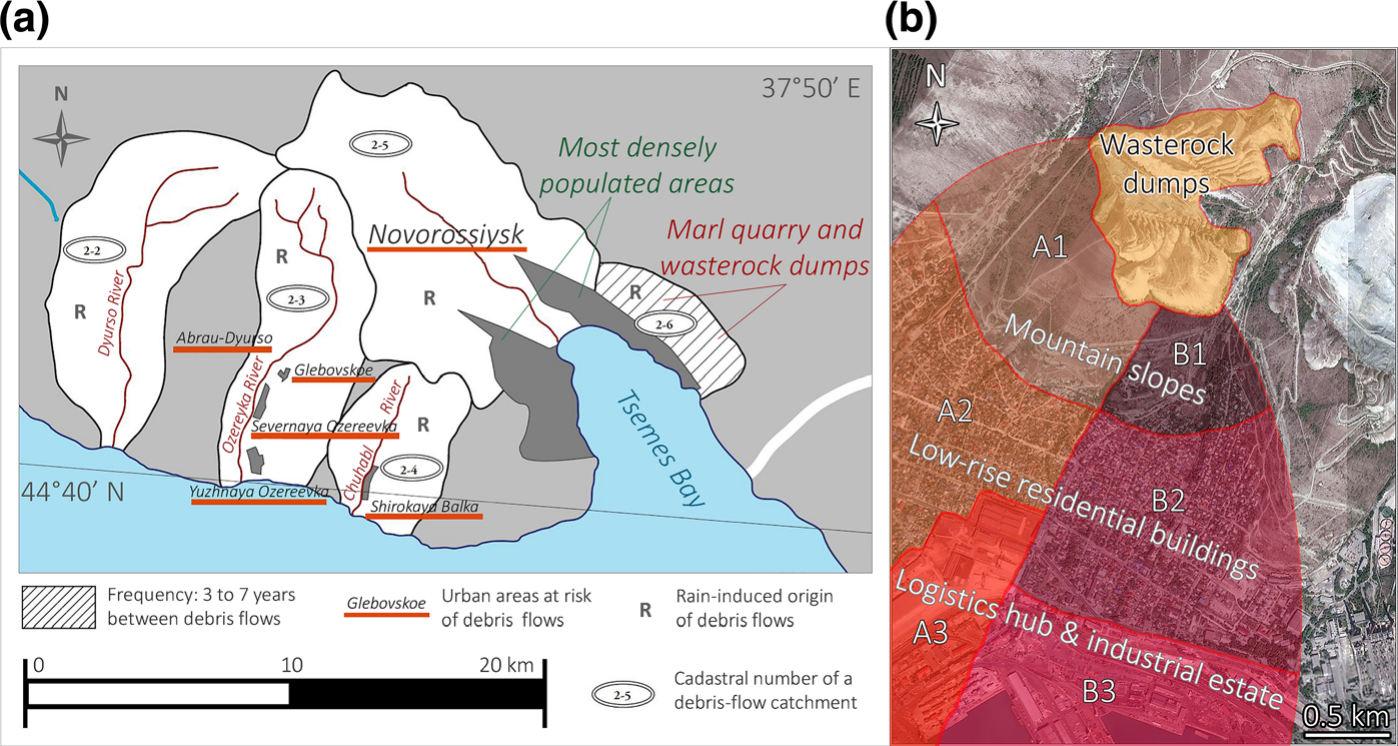
**a** Map of debris flow catchments in the vicinity of Novorossiysk (modified after Perov et al., 2012); **b** Land zoning scheme of the debris-flow-risk area adjoining the dumps in the Novorossiysk industrial agglomeration (based on the *Yandex* satellite image). *Areas A1–A3 and B1–B3 are the key possibly affected zones by a debris flow from the unstable mine-waste storage site*

A debris flow in the dry subtropics on the Black Sea coast of the Caucasus can emerge if a large volume of water gets caught in a small area due to (i) annual storm rainfall precipitation and (ii) waterspouts, i.e., vertical water funnels. Analysis of long-term precipitation observations in the Novorossiysk region (Fig. 2) allows the conclusion that the risk of debris flows is greatest in January, February, November, and December. A major share of tornadoes is formed in the summer, and part of waterspouts comes to land with the "discharge" of large water volumes (Kalmykova & Shershakov, 2016; Simeonov et al., 2013). Most waterspout cases in Northern Eurasia are reported in the Black and Baltic Seas. Over the land, the Black Sea tornadoes do not lose force at a distance of several kilometers from the seashore (Kahraman & Markowski, 2014). A great number of fatalities are characteristic for severe convective events on the Black Sea coast that are accompanied by landfalling waterspouts, heavy showers, and flash floods; e.g., the events of August 8, 2002, August 1, 1991, and October 15, 2010, resulted in 62, 35, and 26 fatalities, respectively. Specifically, during the event of 2002, observers detected salty water in a rain gauge at the Novorossiysk meteorological station that came when waterspouts occurred that day (Chernokulsky et al., 2020). In general, the Novorossiysk agglomeration is a zone of increased debris flow risk on the scale of the entire Black Sea coast of the Caucasus (Fig. 5a).

##### Unconsolidated rocks

In the vicinity of Novorossiysk, potential debris flows can consist of soils and loose sediments overlapping parent rocks. At the dumps, those are cretaceous-age marls in a thick flysch stratum. Their breakage by plentiful cracks in the near-surface layer contributes to active physical weathering and accumulation of clastic material, which can become a solid component of a flow (Rodriguez et al., 2015). If rocks of a 5-cm layer are dragged by a flow from a catchment area of 3 km^2^, a stream volume of 150,000 m^3^ will be formed. Up to 65% of the loose rock mass in a debris flow can consist of 1–10 cm-sized fragments of marl stored in the dumps. Currently, the distance from the dumps to the cliff edge is about 30 m. This is enough to ensure that under normal conditions the debris practically does not slide down the slopes and does not become a part of a flow. However, waterspouts or prolonged rainfalls may change the situation radically, and hundreds of cubic meters of debris may rush into the city with a flow. The quickly weathered debris brought out by the flow to the housing estate can significantly change the geochemical patterns of the soils of garden plots.

This threat required an assessment of the risk magnitude, taking into account the probability of a debris flow and possible implicated damage. The calculations for scenarios of debris flows in the key possibly affected areas allowed evaluation of economic losses in case of land degradation, destruction of privately owned houses and subsidiary holdings, railways and the urban road system, including the Russian federal highway, and partial damage or demolition of port facilities. Zoning of the territory by the degree of man-caused (technogenic) emergency requires calculating the risk of a debris flow: *R*_df_ = ΣΣ *λ*_*i*_ × *P*_*ij*_ × *Y*_*ij*_ (  /year or €/year), where *i* stands for a number of possible emergency scenarios; *j* is the a number of harmful impact types for each scenario; *λ*_*i*_ is the frequency of each scenario per year; *P*_*ij*_ is the probability of each harmful impact type; and *Y*_*ij*_ is the amount of loss caused by each harmful impact type and each scenario,  or €.

##### The A-scenario

In the case of the first considered emergency scenario, seawater discharges into the gully **A1** (Fig. 5b). In this situation, the disturbed land area **A2** will measure up to 200 thousand square meters ( 100 million), up to 200 low-rise residential buildings with adjacent outbuildings and subsidiary holdings will be destroyed ( 900 million). The total length of the destroyed dense road system in the area of the privately owned houses can be up to 10 km ( 20 million). Destruction of the Russian federal M4 Don highway and railroad tracks in the lower **A3** zone ( 8 million) will lead to a complete blockage of on-land transport routes to the Novorossiysk commercial seaport. Engineering port facilities, load-handling yards, and warehouses (**A3**) are also under threat of partial or total demolition ( 600 million) in case of a possible debris flow.

##### The B-scenario

A debris flow from the watershed **B1** will disturb the area of up to 300 thousand m^2^ ( 150 million) and destroy up to 300 households ( 1350 million) and up to 15 km of roads located on the **B2** plot ( 25 million). The area of the disturbed downslope **B3** zone, which includes the highway, railroad ( 12 million), and port facilities ( 800 million), is 1.5 times larger compared to the **A3** zone.

The P_A1_ probability is 30%; P_B1_ is 70%; the proportion is based on the ratio of the volumes of mine spoils stored in each of the catchments. The waterspout discharge frequency was not evaluated separately; however, we could consider such a situation as occurring with a probability of no higher than *n* × 10^–3^ per year (Kalmykova et al., 2019; Vilibić et al., 2018). On average, 15 debris flows occur in this region annually (Barinov et al., 2011; Shnyparkov et al., 2013). The frequency λ for both scenarios is 15 × 10^–3^ per year. The risk in the A-scenario is: *R*_A1_ = Σ *λ*_A1_ × *P*_A1_ × *Y*_A1_ = 15 × 10^–3^ × 0.3 × 100 million + 900 million + 20 million + 8 million + 600 million) = 7.33 million = €94.5 thousand. The risk in the B-scenario is: *R*_B1_ = Σ *λ*_B1_ × *P*_B1_ × *Y*_B1_ = 10.52 million = €135.5 thousand. The calculated cost of the risk damage takes into account estimates on debris flow probability per year and damage inflicted by emergency events, not the absolute damage.

The real case happened in 2002 when a severe flash-flood hit Novorossiysk (precipitation rate was 362 mm/day), around 8 thousand houses were damaged. Four waterspouts were observed; one of them came to the land and enforced the flood by bringing an additional amount of water; saltwater was observed in a rain gauge at the weather station. Total damage was estimated at 2 billion– 184 × 10^–4^% of the Russian GDP (Chernokulsky et al., 2020).

#### Risk clustering

According to the research findings, the following set of factors is taken into account in the Novorossiysk agglomeration when classifying the risks posed by the dumps.

(i) *Factors of the impact on the atmosphere, hydrosphere, lithosphere, and biota.* The dumps are a source of dusting, which is caused by the fugitive emission of fine fractions of rock eluvium from an area of over 150,000 m^2^. Being settled by the dust from the urban air, pollutants create pedochemical anomalies in the soil cover, from which heavy metals and metalloids pass to plants as a result of natural migration (Spijker et al., 2011). With the surface runoff, pollutants migrate from the soil to the water area, where the hydrochemical anomaly is inextricably linked with the biogeochemical one (Moore, 2006). In addition, the discharge of seawater by a waterspout over the dumps can result in a debris flow, posing a threat to the downstream port and onshore facilities.
(ii) *Factors of the impact on human health*. Dust directly affects the human respiratory system and the process is more hazardous after the sorption of pollutants (Ding & Hu, 2014). In conditions of the arid climate, natural air purification by precipitation does not occur, which worsens the medical and environmental situation.
(iii) *Factors of the impact on the social environment and economic security*. When dumping the spoils, the area of land suitable for agricultural use was reduced, the natural landscape changed, erosion processes began, and adjacent land productivity decreased. The dumping area is unattractive for potential investments as long as the unstable dumps pose a geotechnical threat to any facilities. As a result of this, significant areas of one of the city’s districts located near roads and railways, as well as the port, were allocated exclusively for low-rise private buildings with undeveloped urban infrastructure (Nevskaya et al., 2019).
(iv) *Factors of the psychological impact due to the presence of a danger source.* In addition to the health effects, the combination of the debris flow threat, constant air pollution, and disturbed terrain, which has lost its aesthetic value, generally negatively affects the psychological health of citizens.
(v) *The economic costs of establishing and maintaining risk at an acceptable socially conscious level*. Expenditures consist of both environmental and economic damage and the costs of ensuring the safety of dumps. As the actual technogenic impact increases, the costs of compensating for the damage increase.

The proposed approaches to the ranking of technogenic areas and assessment of eco-risks provide the basis for the development of actions aiming at the reduction of ecological hazard of the mine dumps.

### Risk abatement

#### Determining the focal area of reclamation

The threat of dust spread and debris flow can be reduced by reclamation, ensuring the stability of the dumps. In the meantime, mine spoils in the Novorossiysk industrial agglomeration have a certain potential for use after reclamation, which has been repeatedly confirmed by the successful recovery of similar disturbed lands (Cooke & Johnson, 2002; Doley et al., 2012). To choose the direction of reclamation, we analyzed alternative types of land use that contribute to maintaining the sustainability of the created ecosystem and do not violate the ecological state of the environment. The speed of land restoration, cost reasonableness, and long-term stability with minimal maintenance are the central issues (Bangian et al., 2012; Bradshaw, 1990). To choose the focal area of reclamation, one should be guided by several indicators: (i) how many landscape components are to be changed to restore the original natural ecosystem or create a new one; (ii) how much resources will be required to maintain the stability of the created landscape, viz. amendment, irrigation, cultivation, etc.; (iii) what is the difference in resource requirements between undisturbed and reclaimed territories; (iv) how the created landscape will affect the adjacent natural and technogenic ecosystems. In recent years, experts have created classifications that describe all the possible options for re-involving land in the economy: agricultural zones and water bodies for drinking, cultural, or fishery purposes are possible if the soil meets sanitary and hygienic standards; forest utilization lands are to meet less strict requirements (Kovyazin & Romanchikov, 2018); in certain cases, space can be used in construction and recreational purposes; it is also possible to use the territory for landfilling (Bangian et al., 2012; Masoumi & Rashidinejad, 2011; Narrei & Osanloo, 2011; Soltanmohammadi et al., 2010).

Given the environmental complications of the abandoned mine spoils, the rational reclamation must ensure the stability of bulk masses by application of anti-erosion coatings and the cultivation of soil-holding forest stands. The revitalized land is to become an ecologically balanced forest, which is part of the public green complex, the total area of which in the Novorossiysk industrial agglomeration should be at least 6 km^2^ to meet the recommended proportion of 20 m^2^ per inhabitant (Pivnyak et al., 2011).

#### Engineering reclamation

Two types of dump surfaces are described: (i) subhorizontal leveled surfaces that measure 45,000 m^2^ with a grade not exceeding 15° and (ii) slopes with a grade of 15–45° over the area of 105,000 m^2^. First-type areas do not require any special engineering preparation of the surface before planting soil-stabilizing plants. On the contrary, the biological stage of slope reclamation is only possible after the stabilization of their soil cover. The performed comparative study of possible ways to stabilize the slopes (Garbarino et al., 2018) showed that the optimal result in conditions of the Novorossiysk industrial agglomeration is achieved by covering a slope surface with erosion-control geosynthetics, a fiber-structured rolled material consisting of several layers of extruded polypropylene meshes, superimposed on each other and mechanically or thermally bound by a polypropylene yarn (Table 7). A large number of voids are needed to create the best conditions for grass plantation in case of the hydroseeding of a mixture of improvers and seeds of herbaceous plants, as well as protection against erosion and to act as a filter, preventing the washout of fine soil particles. The following sub-steps are to be taken.

**Table 7:**
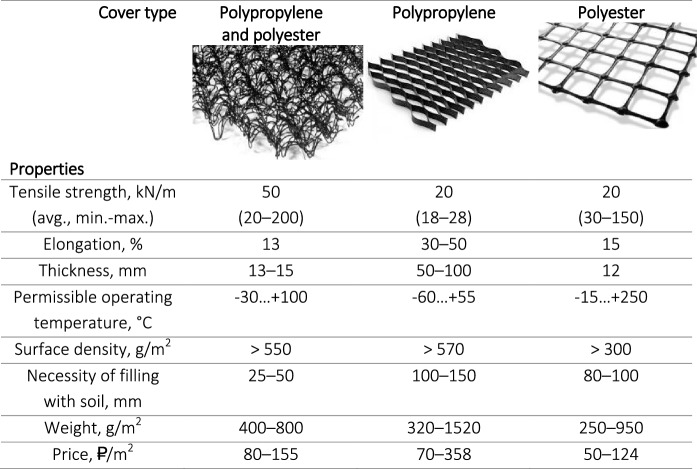
Comparative characteristics of geosynthetics (based on the *miakom.ru* source data)

**Fig. 6:**
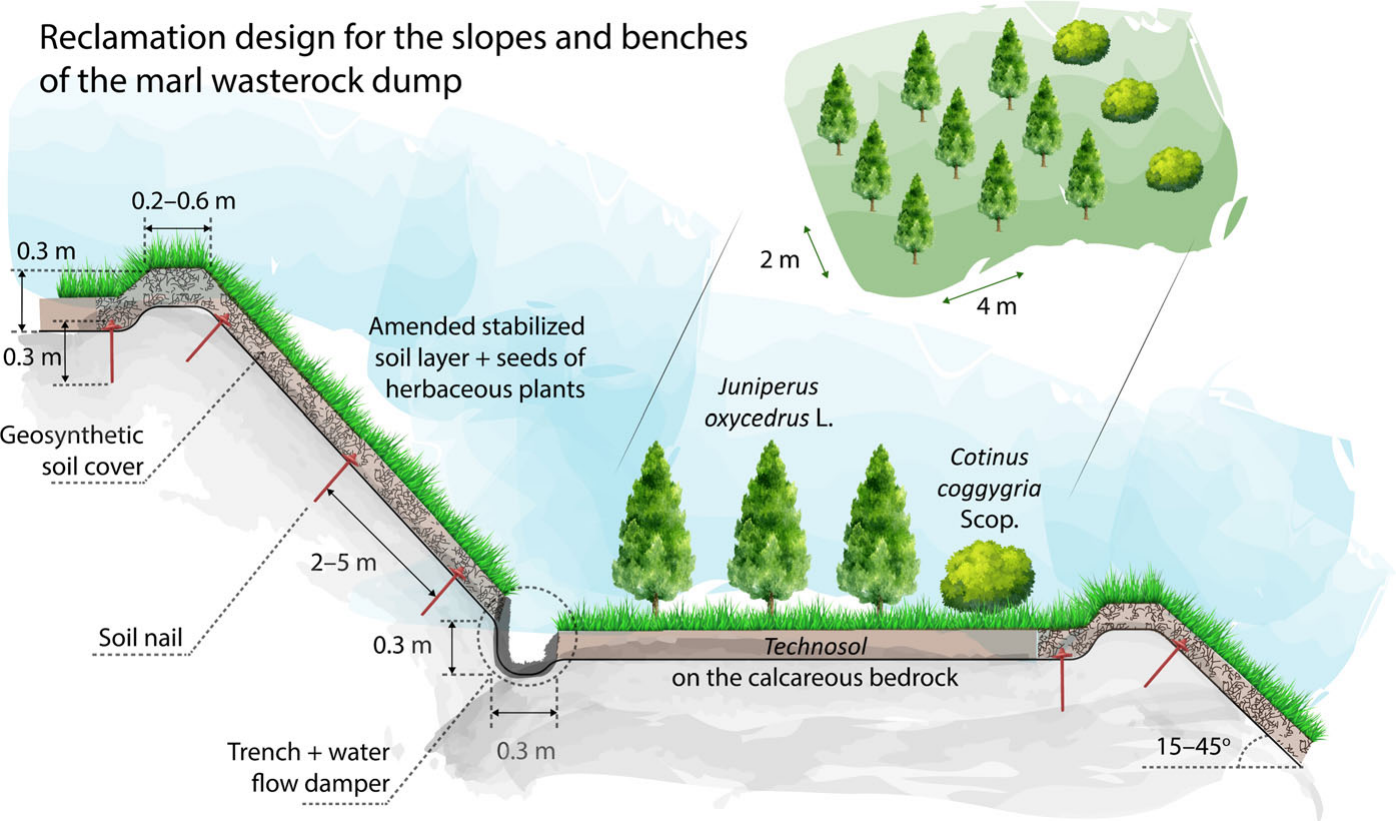
Engineering and biological reclamation: scheme of woody species plantation at the dump benches (smoke trees, 25%, and cades, 75%) and slope stabilization measures

*Surface leveling* of the terraces below and above the reinforced slope using graders prior to applying geosynthetics. Wheeled equipment should be used, as it compacts the soil less than crawler machines do (Ghose, 2005). The existing Technosols and primary associations of herbaceous vegetation provide minimal sodding sufficient for planting at the biological stage of reclamation. To plant the selected tree species, 4,000 pits with dimensions of 0.6 × 0.6 × 0.6 m will be needed per 10,000 m^2^. *Trenches and berms for fixing the geosynthetics cover.* Digging of trenches of a trapezoidal section is necessary with a depth and a width of 0.3 m at a distance of 0.2–0.6 m from the edge of the subgrade above the slope. It is also necessary to install a similar-sized water flow damper at the base of the slope to implement a drain and to prevent the terrace erosion. All the works are to be conducted using an excavator. *Erosion-control geosynthetics* will be laid manually from the upper slope part with its embedment in previously prepared trenches by soil nails with a diameter of 3–5 mm and a length of 30 cm with a bent upper and pointed lower ends made of on the spot (Fig. 6). Trenches are then filled with soil and compacted. Adjacent sheets are laid in parallel with an overlap of at least 0.2 m and fixed by soil nails at 2–3 points across the width of the roll 4–6 m along its length. *The soil cover is applied* over the fiber-structured geosynthetics using excavators from top to bottom, leveling and compaction of the soil are carried out manually.

The cumulative expenditures of the engineering reclamation account for €150.50 thousand and include (€, thousands): capital costs (137.67), materials costs (9.05), salary costs (2.09), social fund payments (0.63), amortization (0.56), and miscellaneous spending (0.50).

#### Biological reclamation

Phyto-reclamation measures are necessary to improve the physical, chemical, and biological properties of the soil. The main objective of this stage is to create productive land, consolidate the surface soil layer with the root system of plants, develop a grass stand, and prevent the water and wind erosion of soils on the dump slopes (Conesa et al., 2007). The local conditions require the selection of drought-resistant, fast-growing grasses that can grow on soils with low nutrient content (Caravaca et al., 2002). However, reclamation should be carried out using plants selected not only because of the ability to grow under adverse conditions of dumps but also because of their amending potential (Madejon et al., 2006). The possibility of using plants atypical for existing communities requires careful consideration since cultivated exotic species can become pests to regional phytocenoses. Indigenous species are preferred over introduced ones because they organically interact with the ecosystem and are adapted to climatic conditions (Chaney et al., 2007). The discussion within *the Working Group on Land Reclamation, Environmental Protection, and Best Available Techniques (BAT) in Mining*, a part of *the Russian-German Raw Materials Forum* (Working, 2020), resulted in the ranking of technique efficacy for creating a grass cover (Table 8).

**Table 8:** Comparative expert assessment of grass coverage techniques (scoring by points, 1 corresponds to the lowest value of a parameter and 3 to the highest)

Parameter	Seeding-down	Turf rolling	Hydroseeding
Labor intensity	3	2	1
Running speed	1	2	3
Work season	Spring and fall	April to November	
Costs of surface preparation	3	2	1
Costs of grass cover arrangement	2	3	1
Costs of grass cover maintenance	3	2	1
Irrigation frequency	3	2	2
Grass canopy stability	2	3	3
Soil erosion control	1	3	3
Resistance to flushing from a slope	Up to 20°	Up to 45°	Up to 70°
Decorative appearance	1	3	2

To sow a community of herbaceous plants on the slopes of mine spoils, the use of hydroseeding technology is necessary. To perform the work, it is necessary to use a hydroseeding machine, which mixes all the components of the solution and delivers the blend through a hose and spray it under high pressure. After a few hours, the applied solution gets dry. A crust emerges on the soil surface, protecting the seeds from washing away by atmospheric precipitation, blowing off by the wind, and being eaten by birds. Optimum conditions for seed germination are created under the crust. The technology involves the use of an aqueous solution that is sprayed over the site and includes the following components.

(i) *Grass seeds*. At the first stage of reclamation, it is advisable to use perennial grasses, mainly cereals. These plants have high productivity, quickly forming an organic mat, protecting the surface from wind and water erosion. Besides, cereals are undemanding to soil fertility and most species tolerate a lack of moisture. It is optimal to use species common in the Northwest Caucasus, which have a good ability to form soddy litter: wheatgrass (*Agropyron cristatum*), beardgrass (*Bothriochloa ischaemum)*, and tussocks (*Brachypodium* spp.). The grasses are resistant to drought and lack of nutrients and impede the process of soil degradation by extensive fibrous root systems, becoming a cradle of a stable vegetation cover (Hao et al., 2004; Ivshina et al., 2014; Shu et al., 2002). As the herbaceous community develops, soil conditions are changed, e.g., the content of organic matter increases, the density decreases, the pH is neutralized, and mineral nutrients are accumulated in the humus horizon in an accessible form. Root systems absorb hard-to-reach nutrients; herbs accumulate the necessary compounds for growth and return them to the soil during decay (Li, 2006; Mendez & Maier, 2008; Zhuikova et al., 2017).
(ii) *Amenders*. Ameliorants brought with an aqueous solution improve the chemical composition of the soil, ensure the rapid formation of the root system, and contribute to the growth of the grassy layer. A complex water-soluble fertilizer is required, the composition of which in the ratio 1:1 includes granules of immediate action and granules in a polymer shell. The nitrogen content should be at least 20%, phosphorus—25%, potassium—5%. During the next 2–3 years, in spring or fall, at least 50 kg of fertilizers should be applied per hectare. Site care is to be taken until the grass covers 60–70% of the slopes. Wood chips, compost, and treated manure contribute to the development of grassy communities that improve soil texture and increase cation exchange and water-holding capacity. These materials also serve as fertilizers with a slow release of nutrients and a microbiological inoculant containing live cultures of plant-beneficial microorganisms (Jordan et al., 2002; Tordoff et al., 2000). Also, wood chips increase plant survival rates and are second only to the humus horizon of the soil in terms of N, P, and K accumulation (Sheoran et al., 2009).
(iii) *Hydrogel* for the accumulation of moisture in a bound form, which will be absorbed by plants in between periods of watering. As a base, polyacrylamide or another polymer with an accumulating capacity of at least 0.1 l of water per 1 g of dry matter should be used.
(iv) *Mulching material* based on wood sawdust, suitable for coating rarely cultivated and not tilled soil (with a decomposition period of at least one year), which also indicates the areas where the works have been conducted and thus allows even application of the solution.
(v) *Organic gluten* as a particle binder.

*Woody plants* at the terraces will improve the quality of the cultivated soil through such processes as reduction of nutrient loss throughout erosion and leaching; moisture accumulation; stabilization of the alkaline–acidic conditions; and raise in the rhizosphere biological activity (Androkhanov & Kurachev, 2009; Coates, 2005; Mertens et al., 2007). Moreover, trees create a self-sustaining fertile humus horizon of the soil, while absorbing nutrients from deep horizons when the roots penetrate deeper than the grassy vegetation can reach (Padmavathiamma & Li, 2007; Pulford & Watson, 2003). Deciduous and coniferous litter, together with root secretions, provides the circulation of nutrients in the soil. We suggest two species to be cultivated. Cade, *Juniperus oxycedrus* L. is an evergreen coniferous tree, related to cypress, reaching a height of 5–10 m and a crown diameter of up to 1 m, growing for up to 600 years. Low-height and creeping juniper species are among the most common plants on rocky slopes and rocks in the region. Cade is photophilous, drought tolerant, and unpretentious to soil conditions (Alguacil et al., 2006; Caravaca et al., 2006; Kuter et al., 2014). Smoke tree, *Cotinus coggygria* Scop. is a deciduous strongly branched shrub reaching a height of 5 m and growing for up to 100 years. It is a photophilous and cold-resistant calcicolous plant that does not tolerate excessive soil moisture and grows on dry rocky slopes or calcareous outcrops (Matić et al., 2016). A smoke tree has a highly patulous root system, gives abundant growth from a stump, and can be easily bred by dividing bushes. Its wild growths have erosion and soil protection significance.

**Fig. 7:**
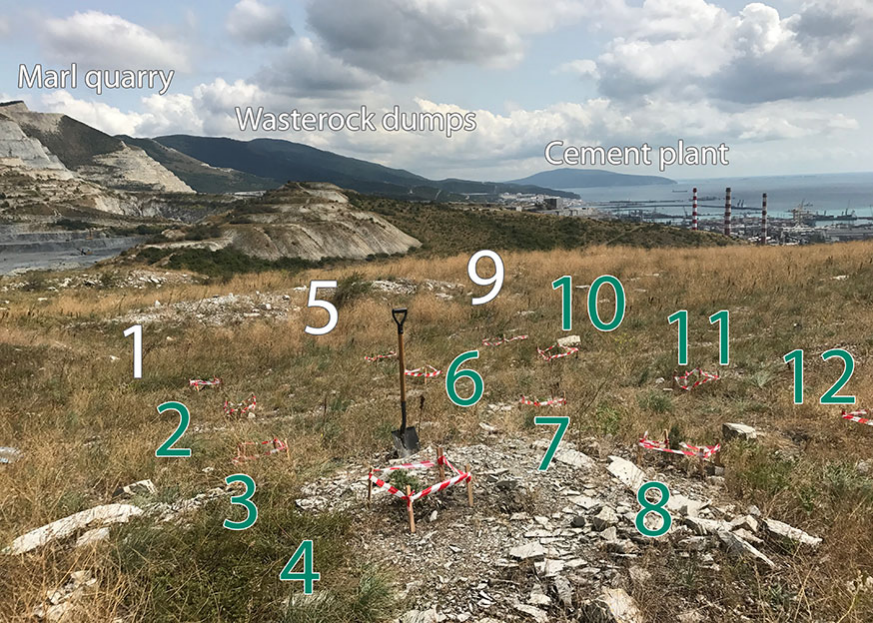
Experimental plantation plot of cades *Juniperus oxycedrus* L. (Nos. 2, 3, 4, 6, 7, 8, 10, 11, and 12) and smoke trees *Cotinus coggygria* Scop*.* (Nos. 1, 5, and 9) at the dump terrace

A small seedling of a tree, previously grown in a nursery, is manually planted in a hole. The cultivation pattern is shown in Fig. 7. The work should be carried out at the end of the summer season, before the beginning of the fall-winter high-rainfall period when the soil is sufficiently wet. On the southern slope of the Markotkh Range, a juniper forest with a smoke-tree story will form an intermittent belt at reclaimed dumps within 100 to 150 m above sea level. The belt boundaries will vary depending on the terrain.

The cumulative expenditures of the biological reclamation account for €54.50 thousand and include (€, thousands): materials costs (49.33), salary costs (2.35), social fund payments (0.71), and miscellaneous spending (2.11). The total reclamation cost of €205 thousand is feasible as it allows the prevention of environmental damage and destruction even in case of the least favorable emergency scenarios.

#### Landscape sustainability

Plantation of a biocenosis including cade and smoke trees on subhorizontal surfaces was carried out on an experimental plot (Fig. 7) and showed the suitability of the trees and shrubs adapted to the dry climatic conditions of the Northwest Caucasus. The works were carried out in the late summer of 2017 at the dump terrace, aiming at the creation of a biogeocenosis close in composition to local plant communities. Cultivated woody plants require watering during the first year after the start of reclamation. The current plant community represented by herbaceous plants will undergo changes as a result of ongoing revitalization. Presently, grasses characteristic of rocky substrates prevail in the biogeocenosis structure: artemisia (*Artemisia caucasica*), milkvetches (*Astragalus* spp*.*), flax (*Linum lanuginosum*), bellflower (*Campanula komarovii*), sages (*Salvia* spp*.*), laserwort (*Laser trilobum*), pyrethrums (*Pyrethrum spp.*), goldendrop (*Onosma taurica*), false brome grasses (*Brachypodium* spp*.*), etc.

**Fig. 8:**
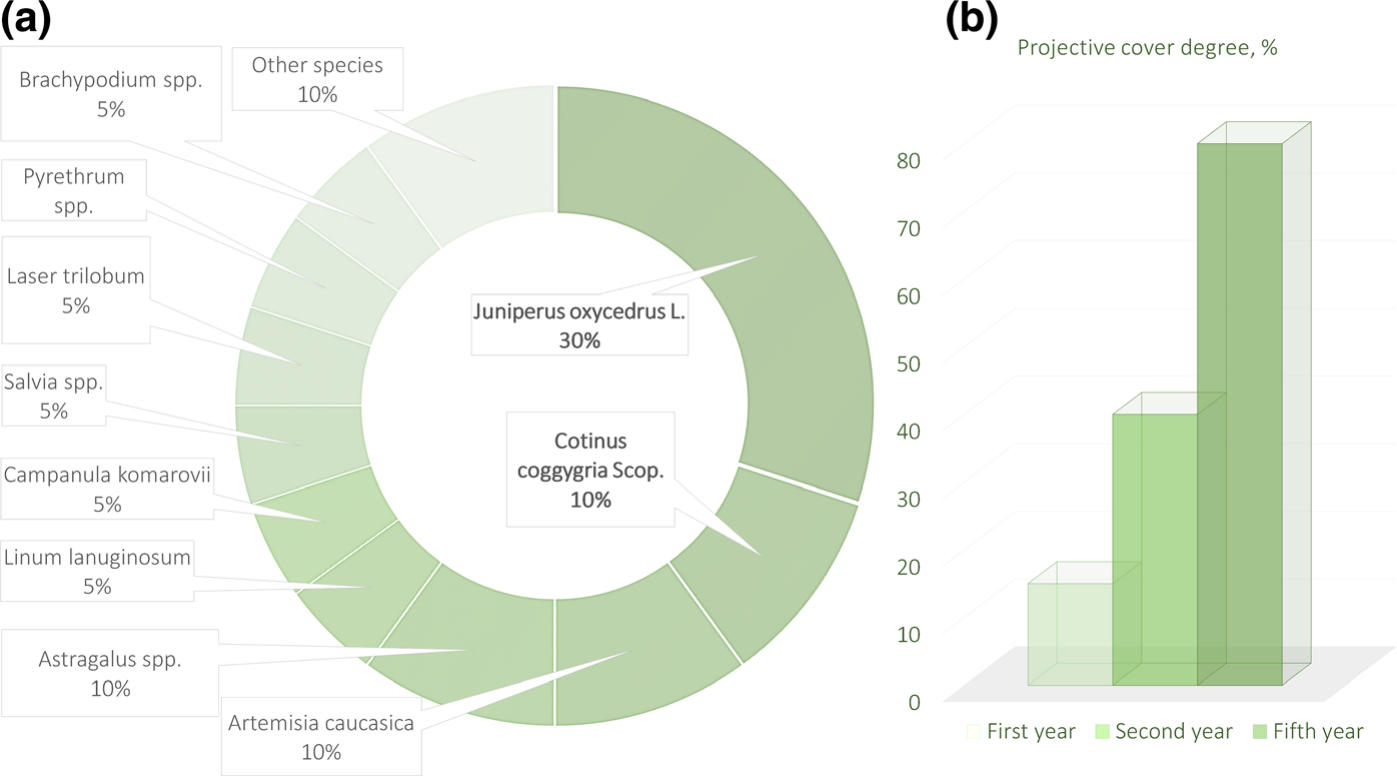
Biological reclamation facts: **a** the current species diversity and the forecasted phytocenosis formed at the dumps in the second year of reclamation (bold letters indicate cades—30% and smoke trees—10%); **b** development of a dense leaf canopy by tree crowns

The survival rate of both tree species planted on thin stony soils is on average 70–80%. In the second year after the launch of the reclamation of the dumps, the phytocenosis structure will significantly evolve on the subhorizontal surfaces (Fig. 8a). A significant share will be taken by tree species, cade (*Juniperus oxycedrus* L.) and smoke tree (*Cotinus coggygria* Scop*.*). The undergrowth of herbaceous plants—legume (Fabaceae) and grasses (Poaceae)—will create the necessary environment for proper maintenance of geochemical cycles of plant nutrients in a certain landscape. This depends on several key factors: (a) accumulation of humus; (b) the presence of nitrogen-fixing plants; and (c) creation of an organic phosphorus reserve (Ghose, 2005). The course of each of these processes can be regulated by the work carried out during reclamation.

- Humic compounds contribute to the metabolic activity of many soil microorganisms that consume carbon as a result of the decomposition of plant and animal matter in the soil; organic C is often a limiting factor in microbial metabolic activity. Cultivation of cereal species on the slopes—wheatgrass (*Agropyron cristatum*), beardgrass (*Bothriochloa ischaemum)*, and tussocks (*Brachypodium* spp.)—supported by fertilization will provide bacteria with enough organic C to stimulate metabolic activity by 80–85% in the first year and raise the average soil moisture by 6–8% (Maiti & Ghose, 2005). The increase in organic C content on the dump terraces will also occur due to the formation of deciduous litter and its humification.
- In mine spoils, nitrogen is one of the top important nutrients limiting plant development. Its fair share required for the development of a phytocenosis is accumulated by the soil during the transformation of atmospheric N, as well as during the subsequent mineralization of dead plants that accumulate organic N. Since the bacteria of the genus *Rhizobium*, capturing atmospheric N, are practically not represented in the underdeveloped Technosols, the reclamation project involves the cultivation of astragals (*Astragalus* spp.), N-fixing legumes. Astragalus that accumulate organic N will create an easily available supply of nutrients in the soil during the decomposition of dead roots (Song et al., 2004). Since N is largely accumulated by the organic matter of soils, the introduction of treated sewage sludge can significantly increase the N content and its availability to roots (Sydnor & Redente, 2002). In addition, organic waste can contribute to moisture retention by the soil.
- The preservation of phosphorus available to plants is difficult at dumps since, with free access of oxygen under supergene conditions, oxidation occurs with the formation of iron oxides that absorb soluble P, which is fixed in forms inaccessible to plants. The tendency increases over time, so it is imperative to provide P-containing organic compounds for the long-term supply of plant needs. Fertilization during reclamation can provide sufficient P over the next several years to support plant growth.

Planting care should be carried out for several years; the soil requires periodic loosening by manual tillage. After the first five years, the projective cover of the mine spoils will increase (Fig. 8b); over the next five years, the tree crowns will create a dense leaf canopy.

Assuming that all proposed reclamation solutions are implemented, the formed vegetation cover will support the natural processes of soil formation at the dumps. The ultimate result of designated steep slope stabilization and tree plantation at subhorizontal sites is the transformation of the technogenic dumps into a biogenic system, characterized by high resistance to erosion and debris flows, as well as the ability to autonomously function without further human intervention.

## Conclusions

The field and laboratory research assessed the complex eco-risk caused by mine spoils in the dry subtropical climate on the Black Sea coast of the Caucasus. The study revealed that in terms of contamination the calcareous dumps pose the indirect *geochemical risk* via dusting and debris flows. Despite the safe levels of macro- and microelements of the waste-rock itself, erosion, deflation, and debris flows are capable of altering the geochemical patterns of the Novorossiysk industrial agglomeration. Firstly, fugitive dust emission from the unreclaimed marl dumps raises the PM2.5 content in the air by a factor of 2.68 on average. High sorption capacity of the fine eluvium results in accumulation of urban emissions by the dust and contributes to the subsequent soil pollution: The proportional share of the territory where the element contents exceed the maximum permissible concentrations in soils makes up from less than 30% (Cu, Zn) to 62% (Pb), creating geochemical anomalies of up to 5 km^2^. The maximum exceedance makes up the association of Pb_19_Zn_9_Cu_2_. According to the state-adopted assessment technique, the inflicted environmental damage to the soil cover accounts already to €2.2 million. Secondly, the unvegetated weathered debris of Technosols can be mobilized by a seawater flow in case of a waterspout, and a debris flow brought out to the housing estate can significantly change the geochemical patterns of the soils by creating technogenic geochemical barriers. The *geotechnical risk* of a debris flow in the key possibly affected areas includes economic losses of land degradation, destruction of privately-owned houses and subsidiary holdings, railways and the urban road system, including the Russian federal highway, and partial damage or demolition of port facilities. The calculated cost of the risk damage takes into account estimates on debris flow probability per year and damage inflicted by emergency events and tots up to €94.5 thousand or €135.5 thousand, according to two scenarios. To *abate the risks* of the dumps cost-effectively, reclamation actions must be taken including soil stabilization on sensitive sites by application of geosynthetic cover, hydroseeding of the mixture of soil improvers and seeds of herbaceous plants on the slopes, and anti-erosion plantation of cades (*Juniperus oxycedrus* L.) and smoke trees (*Cotinus coggygria* Scop.) at subhorizontal surfaces. The experimental cultivation showed the suitability of the selected species adapted to the dry climatic conditions of the Northwest Caucasus at the total cost of €205 thousand. The transformation of the mine spoils into a biogenic system, characterized by high resistance to deflation and debris flows, will prevent dusting and thus indirectly optimize the eco-geochemical state of anthropized urban soils, as well as assure the safety of adjacent residential areas. Restriction of air pollution and protection from potential demolition are vital in the frame of human health maintenance.

## Supplementary Information

Below is the link to the electronic supplementary material.

Supplementary file1 (DOCX 22 kb)

## Data Availability

The authors confirm that the data and materials supporting the findings of this study are available within the article.
