# Supplementary material for: Assessment and abatement of the eco-risk caused by mine spoils in the dry subtropical climate
Source: Environ Geochem Health. 2021 Apr 9;44(5):1581–603. doi: 10.1007/s10653-021-00885-3 (PMC9033738; doi:10.1007/s10653-021-00885-3)
Supplement: Supplementary file 1 — Supplementary file1 (DOCX 22 kb) [file 10653_2021_885_MOESM1_ESM.docx]

**Supplementary material** Particulate matter of ≤1 μm to ≤10 μm diameter triplicate-measured in the air at the height of 1.5 m above ground, mg/m^3^

| **No.** | **Sample** | PM1  average | PM1 minimum | PM1 maximum | PM2.5 average | PM2.5 minimum | PM2.5 maximum | PM4 average | PM4 minimum | PM4 maximum | PM10 average | PM10 minimum | PM10 maximum |
| --- | --- | --- | --- | --- | --- | --- | --- | --- | --- | --- | --- | --- | --- |
| 1 | ND-01-003 | 0.188 | 0.079 | 0.562 | 0.192 | 0.082 | 0.566 | 0.196 | 0.086 | 0.570 | 0.217 | 0.105 | 0.590 |
| 2 | ND-01-004 | 0.205 | 0.080 | 0.951 | 0.209 | 0.083 | 0.955 | 0.213 | 0.086 | 0.959 | 0.235 | 0.107 | 0.980 |
| 3 | ND-01-005 | 0.121 | 0.085 | 0.160 | 0.128 | 0.089 | 0.170 | 0.137 | 0.094 | 0.184 | 0.195 | 0.118 | 0.269 |
| 4 | ND-01-006 | 0.087 | 0.061 | 0.118 | 0.091 | 0.063 | 0.121 | 0.095 | 0.066 | 0.124 | 0.118 | 0.077 | 0.196 |
| 5 | ND-01-007 | 0.054 | 0.041 | 0.069 | 0.057 | 0.043 | 0.071 | 0.060 | 0.045 | 0.074 | 0.076 | 0.053 | 0.103 |
| 6 | ND-01-008 | 0.065 | 0.036 | 0.126 | 0.068 | 0.038 | 0.134 | 0.076 | 0.041 | 0.152 | 0.130 | 0.050 | 0.299 |
| 7 | ND-01-009 | 0.061 | 0.050 | 0.078 | 0.064 | 0.053 | 0.081 | 0.068 | 0.056 | 0.085 | 0.085 | 0.070 | 0.106 |
| 8 | ND-01-010 | 0.098 | 0.070 | 0.289 | 0.101 | 0.073 | 0.292 | 0.106 | 0.077 | 0.297 | 0.131 | 0.098 | 0.324 |
| 9 | ND-01-011 | 0.069 | 0.052 | 0.097 | 0.072 | 0.054 | 0.100 | 0.075 | 0.056 | 0.104 | 0.088 | 0.064 | 0.119 |
| 10 | ND-01-012 | 0.039 | 0.034 | 0.062 | 0.042 | 0.036 | 0.066 | 0.044 | 0.038 | 0.071 | 0.057 | 0.045 | 0.100 |
| 11 | ND-01-013 | 0.055 | 0.048 | 0.073 | 0.057 | 0.051 | 0.076 | 0.060 | 0.053 | 0.080 | 0.074 | 0.062 | 0.102 |
| 12 | ND-01-014 | 0.062 | 0.052 | 0.081 | 0.065 | 0.054 | 0.084 | 0.068 | 0.056 | 0.091 | 0.089 | 0.063 | 0.130 |
| 13 | ND-01-015 | 0.059 | 0.045 | 0.148 | 0.061 | 0.048 | 0.150 | 0.064 | 0.050 | 0.153 | 0.077 | 0.058 | 0.164 |
| 14 | ND-01-016 | 0.058 | 0.033 | 0.123 | 0.060 | 0.035 | 0.125 | 0.062 | 0.037 | 0.127 | 0.071 | 0.042 | 0.135 |
| 15 | ND-01-017 | 0.096 | 0.066 | 0.124 | 0.101 | 0.069 | 0.131 | 0.108 | 0.074 | 0.140 | 0.143 | 0.092 | 0.191 |
| 16 | ND-01-018 | 0.050 | 0.046 | 0.053 | 0.052 | 0.048 | 0.056 | 0.055 | 0.051 | 0.059 | 0.069 | 0.063 | 0.074 |
| 17 | ND-01-019 | 0.149 | 0.058 | 0.567 | 0.153 | 0.061 | 0.573 | 0.157 | 0.064 | 0.581 | 0.181 | 0.080 | 0.620 |
| 18 | ND-01-020 | 0.061 | 0.051 | 0.083 | 0.064 | 0.053 | 0.086 | 0.068 | 0.056 | 0.090 | 0.083 | 0.067 | 0.105 |
| 19 | ND-01-021 | 0.059 | 0.041 | 0.132 | 0.061 | 0.043 | 0.135 | 0.064 | 0.045 | 0.138 | 0.076 | 0.055 | 0.150 |
| 20 | ND-01-022 | 0.039 | 0.035 | 0.051 | 0.041 | 0.036 | 0.053 | 0.042 | 0.038 | 0.055 | 0.047 | 0.039 | 0.062 |
| 21 | ND-01-023 | 0.031 | 0.026 | 0.045 | 0.033 | 0.028 | 0.047 | 0.035 | 0.029 | 0.051 | 0.044 | 0.033 | 0.075 |
| 22 | ND-01-024 | 0.038 | 0.034 | 0.046 | 0.040 | 0.036 | 0.048 | 0.043 | 0.038 | 0.051 | 0.057 | 0.049 | 0.071 |
| 23 | ND-01-025 | 0.051 | 0.040 | 0.103 | 0.054 | 0.042 | 0.106 | 0.056 | 0.044 | 0.108 | 0.071 | 0.052 | 0.123 |
| 24 | ND-01-026 | 0.053 | 0.045 | 0.063 | 0.055 | 0.047 | 0.065 | 0.058 | 0.050 | 0.069 | 0.074 | 0.062 | 0.094 |
| 25 | ND-01-027 | 0.069 | 0.051 | 0.100 | 0.073 | 0.054 | 0.104 | 0.078 | 0.057 | 0.111 | 0.112 | 0.068 | 0.176 |
| 26 | ND-01-028 | 0.054 | 0.045 | 0.062 | 0.057 | 0.047 | 0.065 | 0.060 | 0.050 | 0.069 | 0.077 | 0.061 | 0.096 |
| 27 | ND-01-029 | 0.120 | 0.048 | 0.367 | 0.127 | 0.051 | 0.385 | 0.138 | 0.053 | 0.424 | 0.221 | 0.065 | 0.724 |
| 28 | ND-01-030 | 0.104 | 0.026 | 0.409 | 0.107 | 0.028 | 0.419 | 0.116 | 0.030 | 0.446 | 0.205 | 0.035 | 0.800 |
| 29 | ND-01-031 | 0.043 | 0.023 | 0.119 | 0.045 | 0.025 | 0.123 | 0.048 | 0.026 | 0.131 | 0.065 | 0.030 | 0.225 |
| 30 | ND-01-032 | 0.038 | 0.024 | 0.120 | 0.040 | 0.025 | 0.126 | 0.043 | 0.027 | 0.137 | 0.058 | 0.032 | 0.205 |
| 31 | ND-01-033 | 0.058 | 0.025 | 0.189 | 0.061 | 0.027 | 0.196 | 0.065 | 0.029 | 0.210 | 0.104 | 0.037 | 0.366 |
| 32 | ND-01-034 | 0.042 | 0.022 | 0.104 | 0.043 | 0.024 | 0.106 | 0.046 | 0.025 | 0.108 | 0.053 | 0.033 | 0.117 |
| 33 | ND-01-035 | 0.023 | 0.022 | 0.024 | 0.024 | 0.023 | 0.026 | 0.026 | 0.025 | 0.028 | 0.032 | 0.031 | 0.034 |
| 34 | ND-01-036 | 0.026 | 0.022 | 0.035 | 0.027 | 0.023 | 0.037 | 0.030 | 0.025 | 0.040 | 0.042 | 0.031 | 0.064 |
| 35 | ND-01-037 | 0.029 | 0.024 | 0.036 | 0.032 | 0.026 | 0.038 | 0.034 | 0.029 | 0.042 | 0.046 | 0.037 | 0.059 |
| 36 | ND-01-038 | 0.069 | 0.053 | 0.146 | 0.071 | 0.054 | 0.149 | 0.074 | 0.057 | 0.152 | 0.088 | 0.066 | 0.184 |
| 37 | ND-01-039 | 0.111 | 0.059 | 0.195 | 0.122 | 0.066 | 0.213 | 0.139 | 0.074 | 0.240 | 0.226 | 0.110 | 0.381 |
| 38 | ND-01-040 | 0.046 | 0.036 | 0.081 | 0.049 | 0.039 | 0.084 | 0.052 | 0.041 | 0.088 | 0.070 | 0.053 | 0.110 |
| 39 | ND-01-041 | 0.039 | 0.036 | 0.045 | 0.041 | 0.038 | 0.047 | 0.044 | 0.040 | 0.050 | 0.056 | 0.048 | 0.063 |
| 40 | ND-01-042 | 0.033 | 0.028 | 0.043 | 0.035 | 0.029 | 0.045 | 0.037 | 0.031 | 0.049 | 0.050 | 0.041 | 0.071 |
| 41 | ND-01-043 | 0.049 | 0.039 | 0.088 | 0.052 | 0.042 | 0.093 | 0.057 | 0.046 | 0.101 | 0.084 | 0.064 | 0.165 |
| 42 | ND-01-044 | 0.049 | 0.033 | 0.112 | 0.052 | 0.035 | 0.116 | 0.056 | 0.038 | 0.124 | 0.083 | 0.052 | 0.181 |
| 43 | ND-01-045 | 0.104 | 0.044 | 0.210 | 0.109 | 0.047 | 0.221 | 0.120 | 0.051 | 0.247 | 0.208 | 0.070 | 0.470 |
| 44 | ND-01-046 | 0.083 | 0.057 | 0.108 | 0.086 | 0.060 | 0.111 | 0.090 | 0.065 | 0.116 | 0.117 | 0.093 | 0.151 |
| 45 | ND-01-047 | 0.038 | 0.031 | 0.077 | 0.039 | 0.033 | 0.080 | 0.042 | 0.034 | 0.087 | 0.057 | 0.041 | 0.153 |
| 46 | ND-01-048 | 0.069 | 0.033 | 0.322 | 0.073 | 0.035 | 0.335 | 0.078 | 0.037 | 0.368 | 0.121 | 0.044 | 0.667 |
